# Crystal structure of bis­{μ-4,4′-[1,3-phenyl­enebis(­oxy)]dibenzoato-κ^4^ *O*,*O*′:*O*′′,*O*′′′}bis[(1,10-phenanthroline-κ^2^ *N*,*N*′)zinc(II)] dihydrate

**DOI:** 10.1107/S1600536814018340

**Published:** 2014-09-03

**Authors:** Ya-Ping Li, Li-Ying Han, Julia Ming, Hu Zang, Guan-Fang Su

**Affiliations:** a Department of Ophthalmology, The Second Hospital of Jilin University, 218 Ziqiang Street, Changchun 130041, People’s Republic of China; b Department of Gynaecology, The Second Hospital of Jilin University, 218 Ziqiang Street, Changchun 130041, People’s Republic of China; c St Erik’s Eye Hospital, Karolinska Institute, Polhemsgatan 50, SE-112 82 Stockholm, Sweden; d Department of Orthopedics, The China–Japan Union Hospital of Jilin University Changchun, Changchun 130033, People’s Republic of China

**Keywords:** crystal structure, 4,4′-[1,3-phenyl­enebis(­oxy)]dibenzoate, zinc(II), hydrogen bonding, C—H⋯π inter­actions, π–π stacking

## Abstract

Two 4,4′-[1,3-phenyl­enebis(­oxy)]dibenzoate anions bridge two 1,10-phenanthroline-chelated Zn^II^ cations about a center of inversion to generate the dinuclear title compound, [Zn_2_(C_20_H_12_O_6_)_2_(C_12_H_8_N_2_)_2_]·2H_2_O. The geometry about the Zn^II^ atom is a distorted octa­hedron. In the crystal, the mol­ecules are connected by classical O—H⋯O hydrogen bonds, weak C—H⋯O hydrogen bonds and C—H⋯π inter­actions, forming a three dimensional network. π–π stacking is also observed between aromatic rings of adjacent mol­ecules, centroid–centroid distances are 3.753 (2), 3.5429 (16) and 3.5695 (17) Å.

## Related literature   

For background and related structures, see: Hökelek & Necefouglu (1996 ▶); Necefoglu *et al.* (2002 ▶).
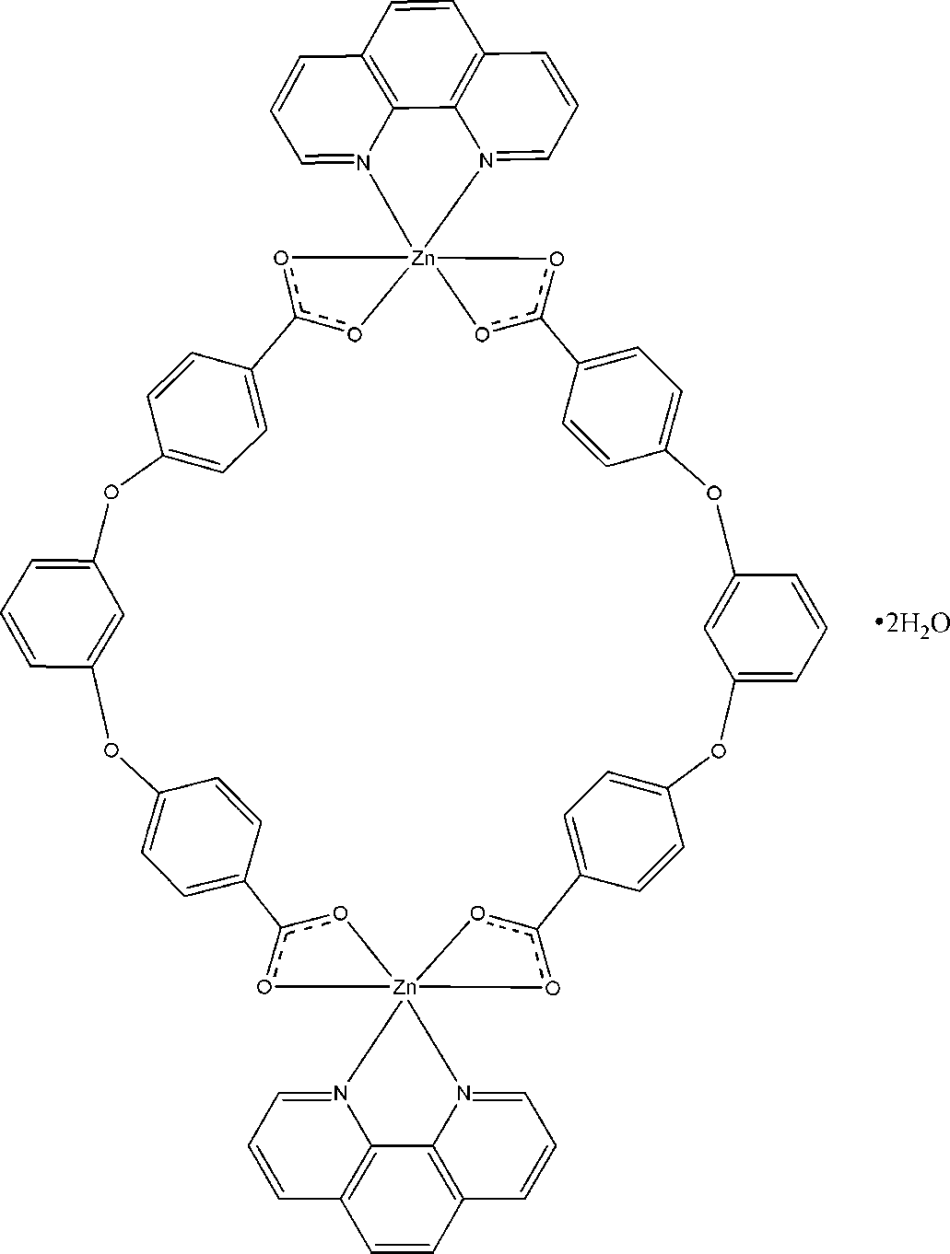


## Experimental   

### Crystal data  

[Zn_2_(C_20_H_12_O_6_)_2_(C_12_H_8_N_2_)_2_]·2H_2_O
*M* *_r_* = 1223.77
Triclinic, 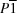

*a* = 10.550 (2) Å
*b* = 11.308 (2) Å
*c* = 12.874 (3) Å
α = 93.210 (4)°
β = 104.225 (4)°
γ = 113.323 (4)°
*V* = 1346.8 (5) Å^3^
*Z* = 1
Mo *K*α radiation
μ = 0.97 mm^−1^
*T* = 293 K
0.28 × 0.23 × 0.21 mm

### Data collection  

Bruker SMART APEXII CCD diffractometer
Absorption correction: multi-scan (*SADABS*; Bruker, 2002 ▶) *T*_min_ = 0.765, *T*_max_ = 0.824
26413 measured reflections
6471 independent reflections
3733 reflections with *I* > 2σ(*I*)
*R*_int_ = 0.073

### Refinement  

*R*[*F* ^2^ > 2σ(*F* ^2^)] = 0.045
*wR*(*F* ^2^) = 0.112
*S* = 1.01
6471 reflections
385 parameters
2 restraints
H atoms treated by a mixture of independent and constrained refinement
Δρ_max_ = 0.22 e Å^−3^
Δρ_min_ = −0.68 e Å^−3^

### 

Data collection: *APEX2* (Bruker, 2002 ▶); cell refinement: *SAINT* (Bruker, 2002 ▶); data reduction: *SAINT*; program(s) used to solve structure: *SHELXS97* (Sheldrick, 2008 ▶); program(s) used to refine structure: *SHELXL97* (Sheldrick, 2008 ▶); molecular graphics: *Mercury* (Macrae *et al.*, 2006 ▶); software used to prepare material for publication: *SHELXTL* (Sheldrick, 2008 ▶) and *publCIF* (Westrip, 2010 ▶).

## Supplementary Material

Crystal structure: contains datablock(s) global, I. DOI: 10.1107/S1600536814018340/xu5810sup1.cif

**Fig. 1.**
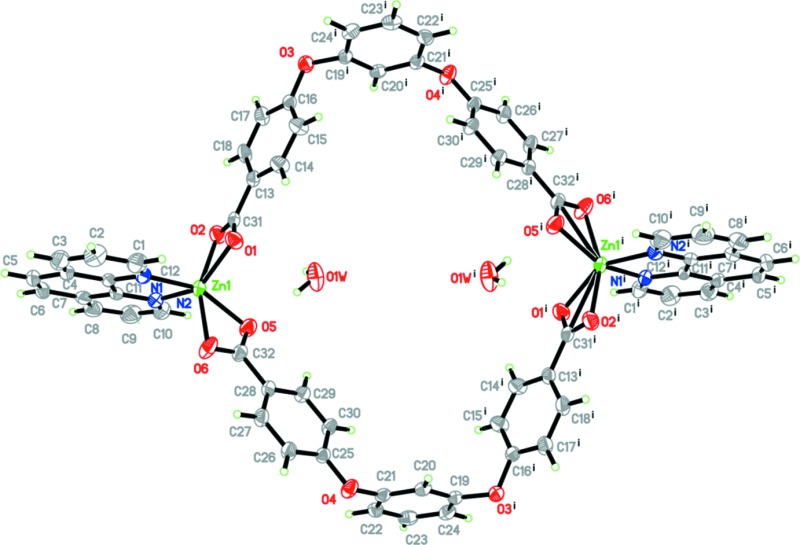
A view of the molecule of the title compound. Displacement ellipsoids are drawn at the 30% probability level. (i) -x, -y, -z.

**Fig. 2.**
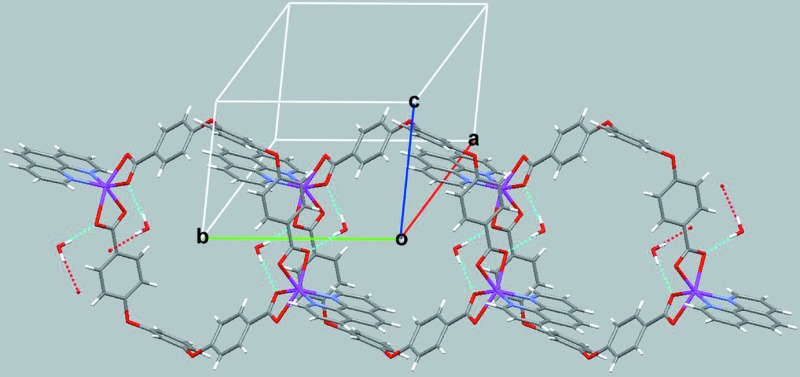
Crystal structure of the title compound with view along the a-axis.

Structure factors: contains datablock(s) I. DOI: 10.1107/S1600536814018340/xu5810Isup2.hkl

x y z . DOI: 10.1107/S1600536814018340/xu5810fig1.tif
A view of the mol­ecule of the title compound. Displacement ellipsoids are drawn at the 30% probability level. (i) −*x*, −*y*, −*z*.

a . DOI: 10.1107/S1600536814018340/xu5810fig2.tif
Crystal structure of the title compound with view along the *a*-axis.

CCDC reference: 1018955

Additional supporting information:  crystallographic information; 3D view; checkCIF report

## Figures and Tables

**Table 1:** Selected bond lengths (Å)

Zn1—N1	2.089 (3)
Zn1—N2	2.097 (3)
Zn1—O1	2.1031 (19)
Zn1—O2	2.2460 (19)
Zn1—O5	2.1061 (19)
Zn1—O6	2.231 (2)

**Table 2:** Hydrogen-bond geometry (Å, °) *Cg*4 and *Cg*6 are the centroids of the C13–C18 and C25–C30 rings, respectively.

*D*—H⋯*A*	*D*—H	H⋯*A*	*D*⋯*A*	*D*—H⋯*A*
O1*W*—H1*A*⋯O2^i^	0.83 (2)	2.06 (2)	2.877 (3)	171 (5)
O1*W*—H1*B*⋯O5	0.84 (2)	2.04 (2)	2.877 (3)	173 (5)
C1—H1⋯O1^ii^	0.93	2.33	3.169 (4)	150
C3—H3⋯O1*W* ^iii^	0.93	2.44	3.332 (4)	161
C5—H5⋯O2^iv^	0.93	2.46	3.256 (4)	144
C8—H8⋯*Cg*6^v^	0.93	2.67	3.543 (4)	156
C10—H10⋯*Cg*4^i^	0.93	2.87	3.726 (5)	154

Symmetry codes: (i) 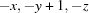; (ii) 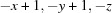; (iii) 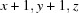; (iv) 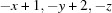; (v) 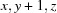.

## supplementary crystallographic information

### S1. Structural commentary

The rational design and construction of coordination polymers based upon assembly of metal ions and multifunctional organic ligands has drawn widespread attentions because of their potential applications as functional materials and intriguing varieties of architectures and topologies (Hökelek & Necefouglu, 1996). The structures of coordination polymers are usually influenced by a multitude of factors such as geometrical and electronic properties of the metal ions employed, coordination abilities of the ligands, the ligand-to-metal ratio, and the use of different solvents (Necefoglu *et al.*, 2002). In this paper, we selected 4,4'-(1,3-phenyl­enebis(­oxy))di­benzoic acid as a linker and 1,10-phenanthroline as a secondary ligand, resulting in the title complex.

In the title compound,[Zn_2_(C_20_H_12_O_6_)_2_(C_12_H_8_N_2_)_2_]^.^2H_2_O, the Zn^II^ atom is surrounded by two N atoms from one 1,10-phenanthroline and four O atoms from two 4,4'-(1,3-phenyl­enebis(­oxy))dibenzoate ligands (Fig. 1). The geometry of the ZnII atom is a distorted o­cta­hedron and the neighboring two Zn^II^ atoms are bridged by two 4,4'-(1,3-phenyl­enebis(­oxy))dibenzoate dianions. Adjacent molecules are connected to the lattice water molecule by hydrogen bonds to form a linear ribbon running along the b-axis of the triclinic unit cell (Fig. 2). Adjacent dimers are further linked through inter­molecular O—H···O hydrogen bonds, leading to a three-dimensional supra­molecular structure (Fig. 2).

### S2. Preparation

The synthesis was performed under hydro­thermal conditions. A mixture of Zn(CH_3_COO)_2_^.^2(H_2_O), (0.2 mmol, 0.044 g), 4,4'-(1,3-phenyl­enebis(­oxy))di­benzoic acid (0.2 mmol, 0.07 g), 1,10-phenanthroline (0.2 mmol, 0.036 g) and H_2_O (20 mL) in a 30 mL stainless steel reactor with a Teflon liner was heated from 293 to 433 K in 2 h and a constant temperature was maintained at 433 K for 72 h, after which the mixture was cooled to 298 K. Colorless crystals of the title compound were recovered from the reaction.

### S3. Refinement

All C—H H atoms were positioned with idealized geometry and refined isotropic with *U*_iso_(H) = 1.2 *U*_eq_(C) using a riding model. The water H-atoms were located in a different Fourier map and were refined with an O—H distance restrained to 0.85 (2) Å and with [*U*_iso_(H) = 1.5 *U*_eq_(O)].

### Crystal data

**Table:**

[Zn_2_(C_20_H_12_O_6_)_2_(C_12_H_8_N_2_)_2_]·2H_2_O	*Z* = 1
*M**_r_* = 1223.77	*F*(000) = 628
Triclinic, *P*1	*D*_x_ = 1.509 Mg m^−^^3^
Hall symbol: -P 1	Mo *K*α radiation, λ = 0.71073 Å
*a* = 10.550 (2) Å	Cell parameters from 6527 reflections
*b* = 11.308 (2) Å	θ = 1.7–22.8°
*c* = 12.874 (3) Å	µ = 0.97 mm^−^^1^
α = 93.210 (4)°	*T* = 293 K
β = 104.225 (4)°	Block, colorless
γ = 113.323 (4)°	0.28 × 0.23 × 0.21 mm
*V* = 1346.8 (5) Å^3^	

### Data collection

**Table:**

Bruker SMART APEXII CCD diffractometer	6471 independent reflections
Radiation source: fine-focus sealed tube	3733 reflections with *I* > 2σ(*I*)
Graphite monochromator	*R*_int_ = 0.073
phi and ω scans	θ_max_ = 28.1°, θ_min_ = 1.7°
Absorption correction: multi-scan (*SADABS*; Bruker, 2002)	*h* = −13→13
*T*_min_ = 0.765, *T*_max_ = 0.824	*k* = −14→14
26413 measured reflections	*l* = −17→16

### Refinement

**Table:**

Refinement on *F*^2^	Primary atom site location: structure-invariant direct methods
Least-squares matrix: full	Secondary atom site location: difference Fourier map
*R*[*F*^2^ > 2σ(*F*^2^)] = 0.045	Hydrogen site location: inferred from neighbouring sites
*wR*(*F*^2^) = 0.112	H atoms treated by a mixture of independent and constrained refinement
*S* = 1.01	*w* = 1/[σ^2^(*F*_o_^2^) + (0.0397*P*)^2^ + 0.2348*P*] where *P* = (*F*_o_^2^ + 2*F*_c_^2^)/3
6471 reflections	(Δ/σ)_max_ = 0.001
385 parameters	Δρ_max_ = 0.22 e Å^−^^3^
2 restraints	Δρ_min_ = −0.68 e Å^−^^3^

### Special details

**Table:**

Geometry. All e.s.d.'s (except the e.s.d. in the dihedral angle between two l.s. planes) are estimated using the full covariance matrix. The cell e.s.d.'s are taken into account individually in the estimation of e.s.d.'s in distances, angles and torsion angles; correlations between e.s.d.'s in cell parameters are only used when they are defined by crystal symmetry. An approximate (isotropic) treatment of cell e.s.d.'s is used for estimating e.s.d.'s involving l.s. planes.
Refinement. Refinement of *F*^2^ against ALL reflections. The weighted *R*-factor *wR* and goodness of fit *S* are based on *F*^2^, conventional *R*-factors *R* are based on *F*, with *F* set to zero for negative *F*^2^. The threshold expression of *F*^2^ > σ(*F*^2^) is used only for calculating *R*-factors(gt) *etc*. and is not relevant to the choice of reflections for refinement. *R*-factors based on *F*^2^ are statistically about twice as large as those based on *F*, and *R*- factors based on ALL data will be even larger.

### Fractional atomic coordinates and isotropic or equivalent isotropic displacement parameters (Å^2^)

**Table:**

	*x*	*y*	*z*	*U*_iso_*/*U*_eq_	
Zn1	0.37437 (4)	0.63248 (3)	0.10279 (3)	0.05945 (15)	
C1	0.6498 (4)	0.7620 (3)	0.0310 (3)	0.0676 (9)	
H1	0.6378	0.6778	0.0079	0.081*	
C2	0.7669 (4)	0.8674 (4)	0.0183 (3)	0.0757 (10)	
H2	0.8330	0.8534	−0.0113	0.091*	
C3	0.7843 (4)	0.9914 (3)	0.0494 (3)	0.0662 (9)	
H3	0.8617	1.0628	0.0406	0.079*	
C4	0.6849 (3)	1.0103 (3)	0.0947 (2)	0.0472 (7)	
C5	0.6945 (3)	1.1366 (3)	0.1300 (2)	0.0567 (8)	
H5	0.7691	1.2110	0.1217	0.068*	
C6	0.5980 (4)	1.1491 (3)	0.1745 (2)	0.0594 (8)	
H6	0.6069	1.2323	0.1972	0.071*	
C7	0.4812 (3)	1.0374 (3)	0.1882 (2)	0.0516 (7)	
C8	0.3761 (4)	1.0452 (4)	0.2320 (2)	0.0702 (9)	
H8	0.3801	1.1263	0.2554	0.084*	
C9	0.2683 (5)	0.9347 (4)	0.2406 (3)	0.0820 (11)	
H9	0.1963	0.9394	0.2679	0.098*	
C10	0.2650 (4)	0.8138 (4)	0.2087 (3)	0.0749 (10)	
H10	0.1916	0.7386	0.2172	0.090*	
C11	0.4700 (3)	0.9131 (3)	0.15552 (19)	0.0445 (7)	
C12	0.5724 (3)	0.8995 (2)	0.10674 (19)	0.0430 (7)	
C13	0.1204 (3)	0.4615 (2)	−0.1993 (2)	0.0421 (6)	
C14	0.1351 (3)	0.3606 (3)	−0.2509 (2)	0.0547 (8)	
H14	0.2029	0.3323	−0.2147	0.066*	
C15	0.0509 (3)	0.3000 (3)	−0.3557 (2)	0.0548 (8)	
H15	0.0596	0.2295	−0.3889	0.066*	
C16	−0.0458 (3)	0.3444 (2)	−0.4107 (2)	0.0420 (6)	
C17	−0.0585 (3)	0.4483 (3)	−0.3621 (2)	0.0549 (8)	
H17	−0.1219	0.4800	−0.4002	0.066*	
C18	0.0237 (3)	0.5060 (3)	−0.2559 (2)	0.0536 (7)	
H18	0.0136	0.5754	−0.2224	0.064*	
C19	0.1702 (3)	−0.1591 (3)	0.5514 (2)	0.0424 (6)	
C20	0.2827 (3)	−0.0663 (3)	0.52396 (19)	0.0423 (6)	
H20	0.3292	−0.0906	0.4798	0.051*	
C21	0.3247 (3)	0.0634 (3)	0.5636 (2)	0.0413 (6)	
C22	0.2588 (3)	0.1018 (3)	0.6293 (2)	0.0486 (7)	
H22	0.2891	0.1898	0.6558	0.058*	
C23	0.1464 (3)	0.0065 (3)	0.6552 (2)	0.0538 (8)	
H23	0.0997	0.0309	0.6991	0.065*	
C24	0.1021 (3)	−0.1237 (3)	0.6173 (2)	0.0501 (7)	
H24	0.0268	−0.1871	0.6361	0.060*	
C25	0.4251 (3)	0.2393 (2)	0.4715 (2)	0.0410 (6)	
C26	0.5499 (3)	0.3294 (3)	0.4562 (2)	0.0510 (7)	
H26	0.6389	0.3326	0.4938	0.061*	
C27	0.5426 (3)	0.4148 (3)	0.3852 (2)	0.0502 (7)	
H27	0.6269	0.4756	0.3750	0.060*	
C28	0.4105 (3)	0.4110 (2)	0.3288 (2)	0.0402 (6)	
C29	0.2878 (3)	0.3202 (3)	0.3449 (2)	0.0458 (7)	
H29	0.1986	0.3161	0.3068	0.055*	
C30	0.2935 (3)	0.2342 (3)	0.4170 (2)	0.0445 (7)	
H30	0.2094	0.1741	0.4281	0.053*	
C31	0.2085 (3)	0.5241 (3)	−0.0847 (2)	0.0453 (7)	
C32	0.4034 (3)	0.5021 (3)	0.2499 (2)	0.0476 (7)	
N1	0.5547 (3)	0.7767 (2)	0.07454 (18)	0.0521 (6)	
N2	0.3636 (3)	0.8020 (2)	0.16621 (18)	0.0570 (7)	
O1	0.3175 (2)	0.50412 (18)	−0.04255 (15)	0.0572 (5)	
O2	0.1746 (2)	0.59693 (19)	−0.03119 (15)	0.0565 (5)	
O3	−0.1312 (2)	0.29119 (17)	−0.51698 (14)	0.0541 (5)	
O4	0.44450 (19)	0.15463 (18)	0.54058 (16)	0.0558 (5)	
O5	0.2821 (2)	0.49319 (19)	0.19589 (16)	0.0621 (6)	
O6	0.5150 (2)	0.5833 (2)	0.23655 (18)	0.0703 (6)	
O1W	0.0278 (3)	0.2886 (2)	0.0473 (3)	0.0911 (8)	
H1A	−0.023 (4)	0.329 (4)	0.044 (4)	0.137*	
H1B	0.106 (3)	0.344 (4)	0.091 (3)	0.137*	

### Atomic displacement parameters (Å^2^)

**Table:**

	*U*^11^	*U*^22^	*U*^33^	*U*^12^	*U*^13^	*U*^23^
Zn1	0.0754 (3)	0.03123 (19)	0.0509 (2)	0.00893 (17)	0.00531 (16)	0.01068 (14)
C1	0.088 (3)	0.055 (2)	0.069 (2)	0.044 (2)	0.0151 (19)	0.0151 (17)
C2	0.076 (3)	0.085 (3)	0.084 (2)	0.048 (2)	0.028 (2)	0.022 (2)
C3	0.057 (2)	0.066 (2)	0.070 (2)	0.0220 (18)	0.0132 (17)	0.0207 (17)
C4	0.0488 (17)	0.0412 (16)	0.0419 (14)	0.0152 (14)	0.0027 (12)	0.0102 (12)
C5	0.062 (2)	0.0364 (16)	0.0520 (16)	0.0099 (15)	0.0012 (15)	0.0089 (13)
C6	0.080 (2)	0.0348 (16)	0.0505 (16)	0.0196 (17)	0.0062 (16)	0.0003 (13)
C7	0.070 (2)	0.0455 (17)	0.0328 (13)	0.0234 (16)	0.0071 (13)	0.0018 (12)
C8	0.098 (3)	0.068 (2)	0.0481 (17)	0.040 (2)	0.0217 (18)	0.0038 (16)
C9	0.097 (3)	0.095 (3)	0.065 (2)	0.044 (3)	0.036 (2)	0.015 (2)
C10	0.071 (2)	0.076 (3)	0.062 (2)	0.011 (2)	0.0261 (18)	0.0199 (18)
C11	0.0540 (17)	0.0386 (15)	0.0307 (12)	0.0151 (14)	0.0025 (12)	0.0046 (11)
C12	0.0528 (17)	0.0298 (14)	0.0350 (13)	0.0143 (13)	−0.0015 (12)	0.0072 (11)
C13	0.0364 (14)	0.0339 (14)	0.0513 (15)	0.0110 (12)	0.0109 (12)	0.0085 (12)
C14	0.0468 (17)	0.064 (2)	0.0547 (16)	0.0335 (16)	0.0014 (13)	0.0053 (15)
C15	0.0541 (18)	0.0627 (19)	0.0516 (16)	0.0369 (16)	0.0036 (13)	−0.0004 (14)
C16	0.0323 (14)	0.0384 (15)	0.0461 (14)	0.0084 (12)	0.0063 (11)	0.0106 (12)
C17	0.0449 (16)	0.0414 (16)	0.0708 (19)	0.0208 (14)	−0.0002 (14)	0.0078 (14)
C18	0.0482 (17)	0.0343 (15)	0.0695 (19)	0.0175 (14)	0.0038 (14)	0.0013 (13)
C19	0.0417 (15)	0.0384 (15)	0.0396 (13)	0.0149 (13)	0.0020 (12)	0.0101 (11)
C20	0.0444 (15)	0.0518 (17)	0.0379 (13)	0.0265 (14)	0.0122 (11)	0.0139 (12)
C21	0.0355 (14)	0.0436 (16)	0.0435 (14)	0.0182 (13)	0.0049 (12)	0.0159 (12)
C22	0.0525 (17)	0.0468 (17)	0.0426 (14)	0.0242 (15)	0.0024 (13)	0.0056 (13)
C23	0.0600 (19)	0.074 (2)	0.0417 (15)	0.0391 (18)	0.0194 (14)	0.0151 (15)
C24	0.0415 (16)	0.0589 (19)	0.0505 (16)	0.0198 (15)	0.0142 (13)	0.0227 (14)
C25	0.0382 (15)	0.0374 (14)	0.0471 (14)	0.0171 (12)	0.0092 (12)	0.0116 (12)
C26	0.0313 (14)	0.0439 (16)	0.0689 (18)	0.0118 (13)	0.0053 (13)	0.0145 (14)
C27	0.0389 (16)	0.0398 (16)	0.0660 (18)	0.0101 (13)	0.0144 (13)	0.0165 (14)
C28	0.0434 (15)	0.0336 (14)	0.0467 (14)	0.0174 (13)	0.0162 (12)	0.0088 (11)
C29	0.0375 (15)	0.0479 (16)	0.0553 (16)	0.0202 (13)	0.0134 (12)	0.0173 (13)
C30	0.0343 (14)	0.0447 (16)	0.0575 (16)	0.0161 (13)	0.0170 (12)	0.0200 (13)
C31	0.0445 (16)	0.0296 (14)	0.0510 (15)	0.0060 (13)	0.0111 (13)	0.0113 (12)
C32	0.0620 (19)	0.0377 (16)	0.0519 (16)	0.0246 (15)	0.0245 (14)	0.0134 (13)
N1	0.0697 (17)	0.0357 (13)	0.0467 (13)	0.0240 (12)	0.0069 (12)	0.0087 (10)
N2	0.0629 (17)	0.0469 (15)	0.0448 (13)	0.0097 (13)	0.0102 (12)	0.0116 (11)
O1	0.0525 (12)	0.0523 (12)	0.0574 (11)	0.0242 (10)	−0.0015 (9)	0.0002 (9)
O2	0.0612 (13)	0.0456 (12)	0.0581 (12)	0.0218 (11)	0.0123 (10)	0.0008 (10)
O3	0.0580 (12)	0.0409 (11)	0.0517 (11)	0.0193 (10)	−0.0013 (9)	0.0098 (9)
O4	0.0370 (10)	0.0532 (12)	0.0769 (13)	0.0192 (9)	0.0116 (9)	0.0317 (10)
O5	0.0547 (13)	0.0552 (13)	0.0721 (13)	0.0229 (11)	0.0083 (10)	0.0278 (10)
O6	0.0598 (14)	0.0667 (14)	0.0943 (16)	0.0249 (12)	0.0353 (12)	0.0475 (13)
O1W	0.0571 (16)	0.0517 (15)	0.154 (3)	0.0151 (12)	0.0307 (16)	0.0002 (15)

### Geometric parameters (Å, º)

**Table:**

Zn1—N1	2.089 (3)	C15—H15	0.9300
Zn1—N2	2.097 (3)	C16—C17	1.369 (4)
Zn1—O1	2.1031 (19)	C16—O3	1.385 (3)
Zn1—O2	2.2460 (19)	C17—C18	1.384 (4)
Zn1—O5	2.1061 (19)	C17—H17	0.9300
Zn1—O6	2.231 (2)	C18—H18	0.9300
Zn1—C32	2.498 (3)	C19—C24	1.374 (4)
Zn1—C31	2.507 (3)	C19—C20	1.377 (4)
C1—N1	1.321 (4)	C19—O3^i^	1.395 (3)
C1—C2	1.389 (5)	C20—C21	1.378 (4)
C1—H1	0.9300	C20—H20	0.9300
C2—C3	1.364 (5)	C21—C22	1.369 (4)
C2—H2	0.9300	C21—O4	1.392 (3)
C3—C4	1.396 (4)	C22—C23	1.379 (4)
C3—H3	0.9300	C22—H22	0.9300
C4—C12	1.393 (4)	C23—C24	1.373 (4)
C4—C5	1.430 (4)	C23—H23	0.9300
C5—C6	1.333 (4)	C24—H24	0.9300
C5—H5	0.9300	C25—C30	1.370 (3)
C6—C7	1.430 (4)	C25—C26	1.378 (4)
C6—H6	0.9300	C25—O4	1.382 (3)
C7—C8	1.391 (4)	C26—C27	1.377 (3)
C7—C11	1.395 (4)	C26—H26	0.9300
C8—C9	1.348 (5)	C27—C28	1.387 (3)
C8—H8	0.9300	C27—H27	0.9300
C9—C10	1.390 (5)	C28—C29	1.369 (4)
C9—H9	0.9300	C28—C32	1.496 (3)
C10—N2	1.335 (4)	C29—C30	1.390 (3)
C10—H10	0.9300	C29—H29	0.9300
C11—N2	1.355 (3)	C30—H30	0.9300
C11—C12	1.428 (4)	C31—O2	1.254 (3)
C12—N1	1.352 (3)	C31—O1	1.258 (3)
C13—C14	1.369 (4)	C32—O6	1.233 (3)
C13—C18	1.382 (3)	C32—O5	1.259 (3)
C13—C31	1.493 (4)	O3—C19^i^	1.395 (3)
C14—C15	1.380 (4)	O1W—H1A	0.829 (19)
C14—H14	0.9300	O1W—H1B	0.842 (19)
C15—C16	1.373 (3)		
			
N1—Zn1—N2	79.28 (10)	C14—C15—H15	120.2
N1—Zn1—O1	95.02 (9)	C17—C16—C15	120.3 (2)
N2—Zn1—O1	142.90 (8)	C17—C16—O3	116.7 (2)
N1—Zn1—O5	150.55 (8)	C15—C16—O3	123.1 (3)
N2—Zn1—O5	104.30 (9)	C16—C17—C18	119.6 (2)
O1—Zn1—O5	98.37 (8)	C16—C17—H17	120.2
N1—Zn1—O6	91.11 (8)	C18—C17—H17	120.2
N2—Zn1—O6	107.25 (9)	C13—C18—C17	120.7 (3)
O1—Zn1—O6	109.50 (8)	C13—C18—H18	119.6
O5—Zn1—O6	59.70 (7)	C17—C18—H18	119.6
N1—Zn1—O2	110.05 (8)	C24—C19—C20	121.0 (3)
N2—Zn1—O2	87.32 (8)	C24—C19—O3^i^	119.7 (2)
O1—Zn1—O2	59.99 (7)	C20—C19—O3^i^	119.2 (3)
O5—Zn1—O2	99.35 (7)	C19—C20—C21	118.3 (3)
O6—Zn1—O2	156.48 (8)	C19—C20—H20	120.9
N1—Zn1—C32	120.63 (9)	C21—C20—H20	120.9
N2—Zn1—C32	109.61 (9)	C22—C21—C20	122.1 (3)
O1—Zn1—C32	104.79 (8)	C22—C21—O4	120.6 (2)
O5—Zn1—C32	30.22 (8)	C20—C21—O4	117.1 (2)
O6—Zn1—C32	29.53 (8)	C21—C22—C23	118.2 (3)
O2—Zn1—C32	128.47 (9)	C21—C22—H22	120.9
N1—Zn1—C31	103.59 (8)	C23—C22—H22	120.9
N2—Zn1—C31	115.33 (9)	C24—C23—C22	121.3 (3)
O1—Zn1—C31	30.07 (8)	C24—C23—H23	119.4
O5—Zn1—C31	101.09 (8)	C22—C23—H23	119.4
O6—Zn1—C31	136.73 (9)	C23—C24—C19	119.1 (3)
O2—Zn1—C31	29.95 (7)	C23—C24—H24	120.4
C32—Zn1—C31	121.22 (9)	C19—C24—H24	120.4
N1—C1—C2	122.6 (3)	C30—C25—C26	120.5 (2)
N1—C1—H1	118.7	C30—C25—O4	124.4 (2)
C2—C1—H1	118.7	C26—C25—O4	115.0 (2)
C3—C2—C1	119.4 (3)	C27—C26—C25	119.9 (2)
C3—C2—H2	120.3	C27—C26—H26	120.1
C1—C2—H2	120.3	C25—C26—H26	120.1
C2—C3—C4	119.4 (3)	C26—C27—C28	120.6 (2)
C2—C3—H3	120.3	C26—C27—H27	119.7
C4—C3—H3	120.3	C28—C27—H27	119.7
C12—C4—C3	117.5 (3)	C29—C28—C27	118.6 (2)
C12—C4—C5	119.1 (3)	C29—C28—C32	121.3 (2)
C3—C4—C5	123.4 (3)	C27—C28—C32	120.1 (2)
C6—C5—C4	120.9 (3)	C28—C29—C30	121.5 (2)
C6—C5—H5	119.6	C28—C29—H29	119.2
C4—C5—H5	119.6	C30—C29—H29	119.2
C5—C6—C7	121.5 (3)	C25—C30—C29	118.9 (2)
C5—C6—H6	119.3	C25—C30—H30	120.6
C7—C6—H6	119.3	C29—C30—H30	120.6
C8—C7—C11	117.4 (3)	O2—C31—O1	120.2 (2)
C8—C7—C6	123.7 (3)	O2—C31—C13	120.2 (2)
C11—C7—C6	118.9 (3)	O1—C31—C13	119.6 (3)
C9—C8—C7	119.8 (3)	O2—C31—Zn1	63.42 (14)
C9—C8—H8	120.1	O1—C31—Zn1	56.89 (14)
C7—C8—H8	120.1	C13—C31—Zn1	175.3 (2)
C8—C9—C10	119.9 (4)	O6—C32—O5	120.4 (2)
C8—C9—H9	120.0	O6—C32—C28	120.4 (3)
C10—C9—H9	120.0	O5—C32—C28	119.2 (2)
N2—C10—C9	122.2 (3)	O6—C32—Zn1	63.15 (14)
N2—C10—H10	118.9	O5—C32—Zn1	57.38 (13)
C9—C10—H10	118.9	C28—C32—Zn1	173.7 (2)
N2—C11—C7	122.9 (3)	C1—N1—C12	118.2 (3)
N2—C11—C12	117.4 (3)	C1—N1—Zn1	128.5 (2)
C7—C11—C12	119.7 (3)	C12—N1—Zn1	113.2 (2)
N1—C12—C4	122.8 (3)	C10—N2—C11	117.7 (3)
N1—C12—C11	117.3 (2)	C10—N2—Zn1	129.4 (2)
C4—C12—C11	119.9 (2)	C11—N2—Zn1	112.8 (2)
C14—C13—C18	118.6 (2)	C31—O1—Zn1	93.04 (17)
C14—C13—C31	121.0 (2)	C31—O2—Zn1	86.64 (15)
C18—C13—C31	120.4 (3)	C16—O3—C19^i^	117.09 (18)
C13—C14—C15	121.2 (2)	C25—O4—C21	118.78 (18)
C13—C14—H14	119.4	C32—O5—Zn1	92.40 (16)
C15—C14—H14	119.4	C32—O6—Zn1	87.31 (16)
C16—C15—C14	119.6 (3)	H1A—O1W—H1B	100 (4)
C16—C15—H15	120.2		
			
N1—C1—C2—C3	−1.5 (5)	N1—Zn1—C32—O5	173.72 (16)
C1—C2—C3—C4	0.7 (5)	N2—Zn1—C32—O5	84.78 (18)
C2—C3—C4—C12	0.6 (4)	O1—Zn1—C32—O5	−81.12 (18)
C2—C3—C4—C5	179.8 (3)	O6—Zn1—C32—O5	175.3 (3)
C12—C4—C5—C6	0.4 (4)	O2—Zn1—C32—O5	−17.9 (2)
C3—C4—C5—C6	−178.8 (3)	C31—Zn1—C32—O5	−53.6 (2)
C4—C5—C6—C7	−0.4 (4)	N1—Zn1—C32—C28	−127.6 (19)
C5—C6—C7—C8	−178.6 (3)	N2—Zn1—C32—C28	143.4 (19)
C5—C6—C7—C11	1.0 (4)	O1—Zn1—C32—C28	−22.5 (19)
C11—C7—C8—C9	−0.5 (4)	O5—Zn1—C32—C28	58.7 (19)
C6—C7—C8—C9	179.1 (3)	O6—Zn1—C32—C28	−126 (2)
C7—C8—C9—C10	1.9 (5)	O2—Zn1—C32—C28	41 (2)
C8—C9—C10—N2	−1.9 (5)	C31—Zn1—C32—C28	5 (2)
C8—C7—C11—N2	−0.9 (4)	C2—C1—N1—C12	0.7 (4)
C6—C7—C11—N2	179.4 (2)	C2—C1—N1—Zn1	−179.0 (2)
C8—C7—C11—C12	178.0 (2)	C4—C12—N1—C1	0.8 (3)
C6—C7—C11—C12	−1.7 (3)	C11—C12—N1—C1	−178.8 (2)
C3—C4—C12—N1	−1.4 (3)	C4—C12—N1—Zn1	−179.44 (17)
C5—C4—C12—N1	179.3 (2)	C11—C12—N1—Zn1	0.9 (3)
C3—C4—C12—C11	178.2 (2)	N2—Zn1—N1—C1	178.5 (2)
C5—C4—C12—C11	−1.1 (3)	O1—Zn1—N1—C1	−38.6 (2)
N2—C11—C12—N1	0.3 (3)	O5—Zn1—N1—C1	78.3 (3)
C7—C11—C12—N1	−178.6 (2)	O6—Zn1—N1—C1	71.1 (2)
N2—C11—C12—C4	−179.3 (2)	O2—Zn1—N1—C1	−98.4 (2)
C7—C11—C12—C4	1.7 (3)	C32—Zn1—N1—C1	71.9 (3)
C18—C13—C14—C15	−2.8 (4)	C31—Zn1—N1—C1	−67.8 (2)
C31—C13—C14—C15	178.1 (3)	N2—Zn1—N1—C12	−1.25 (16)
C13—C14—C15—C16	2.3 (5)	O1—Zn1—N1—C12	141.71 (16)
C14—C15—C16—C17	0.1 (4)	O5—Zn1—N1—C12	−101.4 (2)
C14—C15—C16—O3	178.2 (3)	O6—Zn1—N1—C12	−108.60 (16)
C15—C16—C17—C18	−1.9 (4)	O2—Zn1—N1—C12	81.87 (17)
O3—C16—C17—C18	179.9 (2)	C32—Zn1—N1—C12	−107.81 (17)
C14—C13—C18—C17	1.0 (4)	C31—Zn1—N1—C12	112.47 (17)
C31—C13—C18—C17	−179.9 (2)	C9—C10—N2—C11	0.5 (4)
C16—C17—C18—C13	1.3 (4)	C9—C10—N2—Zn1	−175.5 (2)
C24—C19—C20—C21	−0.5 (3)	C7—C11—N2—C10	0.9 (4)
O3^i^—C19—C20—C21	−176.60 (19)	C12—C11—N2—C10	−178.0 (2)
C19—C20—C21—C22	0.5 (3)	C7—C11—N2—Zn1	177.55 (18)
C19—C20—C21—O4	175.93 (19)	C12—C11—N2—Zn1	−1.4 (3)
C20—C21—C22—C23	−0.5 (3)	N1—Zn1—N2—C10	177.5 (3)
O4—C21—C22—C23	−175.9 (2)	O1—Zn1—N2—C10	93.4 (3)
C21—C22—C23—C24	0.7 (4)	O5—Zn1—N2—C10	−32.4 (3)
C22—C23—C24—C19	−0.8 (4)	O6—Zn1—N2—C10	−94.7 (3)
C20—C19—C24—C23	0.7 (3)	O2—Zn1—N2—C10	66.5 (3)
O3^i^—C19—C24—C23	176.7 (2)	C32—Zn1—N2—C10	−63.6 (3)
C30—C25—C26—C27	−0.3 (4)	C31—Zn1—N2—C10	77.5 (3)
O4—C25—C26—C27	177.1 (3)	N1—Zn1—N2—C11	1.42 (16)
C25—C26—C27—C28	0.0 (4)	O1—Zn1—N2—C11	−82.7 (2)
C26—C27—C28—C29	−0.2 (4)	O5—Zn1—N2—C11	151.45 (16)
C26—C27—C28—C32	−178.6 (3)	O6—Zn1—N2—C11	89.23 (17)
C27—C28—C29—C30	0.8 (4)	O2—Zn1—N2—C11	−109.58 (17)
C32—C28—C29—C30	179.1 (3)	C32—Zn1—N2—C11	120.30 (17)
C26—C25—C30—C29	0.9 (4)	C31—Zn1—N2—C11	−98.66 (18)
O4—C25—C30—C29	−176.3 (2)	O2—C31—O1—Zn1	−3.0 (2)
C28—C29—C30—C25	−1.1 (4)	C13—C31—O1—Zn1	176.39 (19)
C14—C13—C31—O2	−165.4 (3)	N1—Zn1—O1—C31	−108.61 (16)
C18—C13—C31—O2	15.5 (4)	N2—Zn1—O1—C31	−29.8 (2)
C14—C13—C31—O1	15.1 (4)	O5—Zn1—O1—C31	97.69 (16)
C18—C13—C31—O1	−163.9 (3)	O6—Zn1—O1—C31	158.42 (15)
C14—C13—C31—Zn1	55 (2)	O2—Zn1—O1—C31	1.68 (14)
C18—C13—C31—Zn1	−124 (2)	C32—Zn1—O1—C31	127.87 (16)
N1—Zn1—C31—O2	−106.68 (16)	O1—C31—O2—Zn1	2.8 (2)
N2—Zn1—C31—O2	−22.26 (18)	C13—C31—O2—Zn1	−176.6 (2)
O1—Zn1—C31—O2	177.1 (2)	N1—Zn1—O2—C31	82.39 (16)
O5—Zn1—C31—O2	89.53 (15)	N2—Zn1—O2—C31	159.95 (16)
O6—Zn1—C31—O2	146.71 (14)	O1—Zn1—O2—C31	−1.68 (14)
C32—Zn1—C31—O2	113.90 (16)	O5—Zn1—O2—C31	−95.99 (15)
N1—Zn1—C31—O1	76.23 (16)	O6—Zn1—O2—C31	−70.5 (3)
N2—Zn1—C31—O1	160.65 (15)	C32—Zn1—O2—C31	−86.97 (17)
O5—Zn1—C31—O1	−87.56 (16)	C17—C16—O3—C19^i^	−152.2 (3)
O6—Zn1—C31—O1	−30.4 (2)	C15—C16—O3—C19^i^	29.7 (4)
O2—Zn1—C31—O1	−177.1 (2)	C30—C25—O4—C21	−4.9 (4)
C32—Zn1—C31—O1	−63.19 (18)	C26—C25—O4—C21	177.8 (2)
N1—Zn1—C31—C13	34 (2)	C22—C21—O4—C25	−72.3 (3)
N2—Zn1—C31—C13	119 (2)	C20—C21—O4—C25	112.2 (2)
O1—Zn1—C31—C13	−42 (2)	O6—C32—O5—Zn1	4.8 (3)
O5—Zn1—C31—C13	−129 (2)	C28—C32—O5—Zn1	−173.9 (2)
O6—Zn1—C31—C13	−72 (2)	N1—Zn1—O5—C32	−11.0 (3)
O2—Zn1—C31—C13	141 (2)	N2—Zn1—O5—C32	−104.52 (17)
C32—Zn1—C31—C13	−105 (2)	O1—Zn1—O5—C32	105.09 (17)
C29—C28—C32—O6	179.9 (3)	O6—Zn1—O5—C32	−2.66 (16)
C27—C28—C32—O6	−1.8 (4)	O2—Zn1—O5—C32	165.88 (17)
C29—C28—C32—O5	−1.4 (4)	C31—Zn1—O5—C32	135.48 (18)
C27—C28—C32—O5	176.9 (3)	O5—C32—O6—Zn1	−4.6 (3)
C29—C28—C32—Zn1	−57 (2)	C28—C32—O6—Zn1	174.1 (2)
C27—C28—C32—Zn1	121.4 (19)	N1—Zn1—O6—C32	178.62 (18)
N1—Zn1—C32—O6	−1.6 (2)	N2—Zn1—O6—C32	99.50 (19)
N2—Zn1—C32—O6	−90.55 (19)	O1—Zn1—O6—C32	−85.67 (18)
O1—Zn1—C32—O6	103.55 (18)	O5—Zn1—O6—C32	2.72 (17)
O5—Zn1—C32—O6	−175.3 (3)	O2—Zn1—O6—C32	−26.7 (3)
O2—Zn1—C32—O6	166.76 (16)	C31—Zn1—O6—C32	−70.1 (2)
C31—Zn1—C32—O6	131.10 (18)		

Symmetry code: (i) −*x*, −*y*, −*z*.

### Hydrogen-bond geometry (Å, º)

Cg4 and Cg6 are the centroids of the C13–C18 and C25–C30 rings, respectively.

**Table:**

*D*—H···*A*	*D*—H	H···*A*	*D*···*A*	*D*—H···*A*
O1*W*—H1*A*···O2^ii^	0.83 (2)	2.06 (2)	2.877 (3)	171 (5)
O1*W*—H1*B*···O5	0.84 (2)	2.04 (2)	2.877 (3)	173 (5)
C1—H1···O1^iii^	0.93	2.33	3.169 (4)	150
C3—H3···O1*W*^iv^	0.93	2.44	3.332 (4)	161
C5—H5···O2^v^	0.93	2.46	3.256 (4)	144
C8—H8···*Cg*6^vi^	0.93	2.67	3.543 (4)	156
C10—H10···*Cg*4^ii^	0.93	2.87	3.726 (5)	154

Symmetry codes: (ii) −*x*, −*y*+1, −*z*; (iii) −*x*+1, −*y*+1, −*z*; (iv) *x*+1, *y*+1, *z*; (v) −*x*+1, −*y*+2, −*z*; (vi) *x*, *y*+1, *z*.
