# Supplementary figures and images for: Crystal structure of bis­{μ-4,4′-[1,3-phenyl­enebis(­oxy)]dibenzoato-κ4 O,O′:O′′,O′′′}bis[(1,10-phenanthroline-κ2 N,N′)zinc(II)] dihydrate
Source: Acta Crystallogr Sect E Struct Rep Online. 2014 Sep 3;70(Pt 10):m341–2. doi: 10.1107/S1600536814018340 (PMC4257206; doi:10.1107/S1600536814018340)

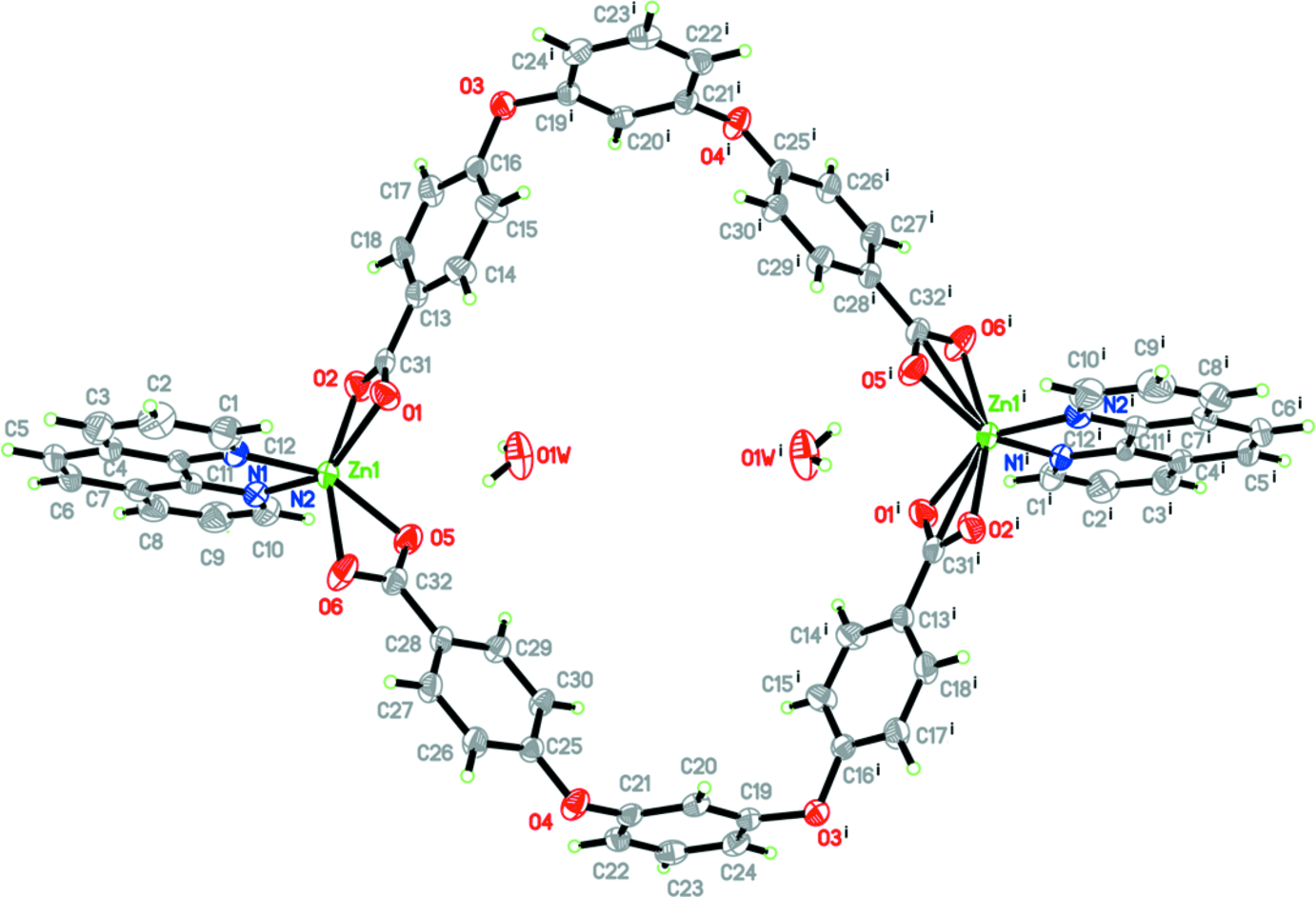

Supplement: Supplementary file 3 [file e-70-0m341-fig1.tif]

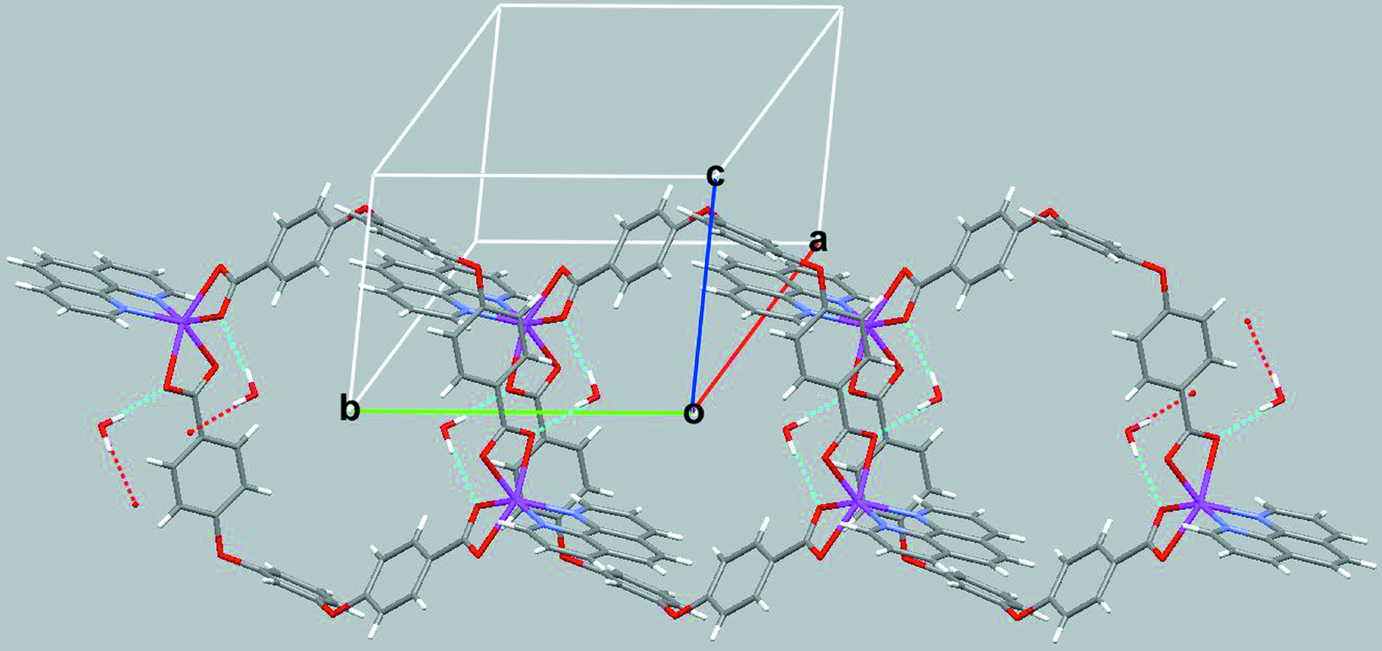

Supplement: Supplementary file 4 [file e-70-0m341-fig2.tif]
